# Job Satisfaction Mediates Associations of Change Fatigue, Organizational Support, and Work Overload with Quiet Quitting Among Healthcare Workers

**DOI:** 10.3390/healthcare14193321

**Published:** 2026-10-06

**Authors:** İbrahim Gün, Erhan Ekingen, Ahmet Yıldız, Juan Gómez-Salgado, Murat Yıldırım

**Affiliations:** 1 Department of Health Management, Faculty of Health Sciences, Batman University, Batman 72060, Türkiye; ibrahim.gun@batman.edu.tr (İ.G.);; 2 Department of Medicine and Public Health, Faculty of Labour Sciences, University of Huelva, 21071 Huelva, Spain; 3 Safety and Health Postgraduate Program, Universidad Espíritu Santo, Guayaquil 092301, Ecuador; 4 Department of Psychology, Ağrı İbrahim Çeçen University, Ağrı 04100, Türkiye; 5 Department of Psychology, Faculty of Arts and Sciences, Haliç University, İstanbul 34060, Türkiye; 6 Psychology Research Center, Khazar University, Baku AZ1096, Azerbaijan

**Keywords:** change fatigue, organizational support, work overload, job satisfaction, quiet quitting

## Abstract

**Background:** In the demanding healthcare environment, change fatigue, lack of organizational support, and work overload have been linked to burnout and disengagement, potentially leading to quiet quitting. This study examined the mediating role of job satisfaction in the relationship between change fatigue, organizational support, work overload, and quiet quitting among healthcare workers. **Methods:** The study used a cross-sectional design, collecting data from 487 healthcare professionals (57.3% females) across various roles, including physicians, nurses, midwives, health officers, and technicians. The research used paper-based questionnaires, including socio-demographic questions and self-report measures for change fatigue, organizational support, work overload, job satisfaction, and quiet quitting. Data collection took place in Türkiye during January and February 2024. Three separate simple mediation analyses were conducted using PROCESS Model 4, with 5000 bootstrap samples and 95% bootstrap confidence intervals. **Results:** Change fatigue, organizational support, and work overload were significantly associated with job satisfaction and quiet quitting. Job satisfaction was also significantly associated with quiet quitting. Furthermore, significant indirect associations of change fatigue, organizational support, and work overload with quiet quitting through job satisfaction were observed. The standardized indirect associations were significant for change fatigue (β = 0.14, SE = 0.01, 95% bootstrap CI [0.11, 0.17]), organizational support (β = −0.11, SE = 0.02, 95% bootstrap CI [−0.15, −0.06]), and work overload (β = 0.13, SE = 0.01, 95% bootstrap CI [0.09, 0.17]). **Conclusions:** These findings suggest that job satisfaction may represent a potentially relevant explanatory pathway linking these workplace factors with quiet quitting.

## 1. Introduction

In the current dynamic and competitive business environment, comprehending employee behaviors and addressing their job-related needs and desires is imperative for organizations aiming to cultivate a productive and stable workforce [1]. Over the past five years, there have been remarkable changes in the field of the business environment, such as shutdowns, lockdowns, and severe economic and financial crises due to COVID-19 [2]. Companies and workers swiftly embraced remote work arrangements, prompting numerous employees to acknowledge the advantages, including flexibility, convenience, and improved work–life balance. Nevertheless, issues such as job and wage disparities, heightened workloads, and role conflicts have also become prevalent in the corporate landscape [3]. During the pandemic, some workers have seen how their jobs could be redesigned remotely or flexibly, while also experiencing virtual burnout and blurred boundaries between work and home. As return-to-office requests began, it inevitably resulted in disappointment and disconnection for many employees. This disconnect led to the “Great Resignation,” where many who could afford to quit their jobs did so. Kehinde et al. stated that “The Great Resignation refers to the unprecedented wave of voluntary quits and job changes that workers across various sectors and countries” [4]. In contrast, quiet quitting represents a form of behavioral withdrawal in which employees remain employed but restrict their efforts to formal job requirements [5]. For employees who are unable or unwilling to resign because of low wages, financial insecurity regarding retirement, limited career alternatives, demanding work conditions, and extended working hours, quiet quitting has emerged as an alternative behavioral response to the Great Resignation. Quiet quitting behavior has served as an alternative for those who cannot or choose not to leave their jobs [6]. A growing number of individuals are increasingly inclined to prioritize investing their energy into their personal lives rather than adhering to the notion of surpassing themselves in the workplace [7]. The majority of workers were compelled to remain at home, except for healthcare workers [5]. Following the pandemic and the phenomenon termed “The Great Resignation”, a novel idea known as “quiet quitting” surfaced initially within social media circles, later garnering attention as mainstream news topics in popular publications, and more recently becoming subjects of discussion within influential think tanks like McKinsey and Gallup [8]. Thereafter, quiet quitting has become a rising concern across societies globally and on various platforms [9]. Certainly, the COVID-19 pandemic has induced social and economic instability, intensifying the strain on numerous individuals and prompting them to reconsider their lifestyle and work practices [7]. The situation is so critical that, according to a study conducted in the USA, up to 47% of healthcare workers plan to resign from their jobs by 2025, and it rises to 90% of nurses [10]. Based on recent Gallup research, 50% of the American workforce is quitting their work [11]. Additionally, according to a recent Wall Street Journal article, 50% of today’s workforce has chosen to limit their commitment to their jobs [12]. Interestingly, this trend seems to be prevalent among young workers, as indicated by an Axios poll revealing that 85% of Gen Z workers find quiet quitting appealing, with 15% already practicing it [13]. Although Mark Boldger initially formalized the term “quiet quitting” to describe employees who limit their engagement at work, the behavior itself is not new and has existed in workplaces for many years [12]. The concept of quiet quitting has gathered widespread description and discussion, especially in social media discourse post-COVID-19. This trend typically entails employees opting to restrict their work efforts to the essential minimum rather than exceeding expectations [14].

Quiet quitting refers to a phenomenon in the workplace where “one does not literally quit one’s job but rather simply does the work that is expected of the position, without going above and beyond what is expected” [11]. Employees who put minimal effort at work due to inadequate organizational support, concerns about wages, job instability, and/or other priorities are termed “quiet quitters” [8]. Additionally, “employees who disengage from their work and organization but do not officially resign are referred to as quiet quitter” [15]. Accordingly, although quiet quitting may overlap conceptually with withdrawal-related constructs, its defining feature in the present study is that employees remain in their jobs while limiting their effort and discretionary contribution rather than actually resigning. Thus, quiet quitting should not be equated with actual turnover or with turnover intention, because leaving the organization—or intending to leave it—is not a necessary defining feature of quiet quitting [16]. With the rapid development of the literature on quiet quitting, factors contributing to quiet quitting are emerging, leading to a better understanding of the concept. Several personal reasons have been pinpointed for quiet quitting behavior, such as employees’ perceptions that they lack the necessary personal resources (e.g., mental and physical energy) to advance professionally or perform their duties effectively [8]. In addition, employees’ desire to maintain a better work–life balance may also contribute to quiet quitting tendencies [5]. Beyond these personal considerations, several organizational factors have been associated with quiet quitting, including feeling neglected by employers, limited professional growth opportunities, a disconnect between organizations and their employees, and growing dissatisfaction and disillusionment with managers [5,13]. Furthermore, organizational environments characterized by inadequate responsiveness to employees’ needs and destructive leadership styles, including narcissistic, psychopathic, Machiavellian, and despotic leadership, may diminish employee motivation and contribute to the emergence of quiet quitting [13]. The phenomenon of quiet quitting, which has emerged as a result of the apparent failures of many contemporary leaders in motivating their employees, is increasingly becoming the response of a growing number of workers [12]. Additionally, studies show that job burnout and workplace bullying are important predictors of quiet quitting [17]. Healthcare workers may be particularly vulnerable to quiet quitting because they face multiple occupational and psychosocial challenges, including infection risk, unfavorable working conditions, economic difficulties and inflation, heavy workloads, wage inequalities, toxic workplace cultures, physical and verbal violence, anxiety, depression, burnout, and work–life imbalance [3]. Moreover, many organizations set high-performance standards without offering adequate compensation, leading to intentions of quiet quitting [15].

Theories such as social exchange, psychological contract, and organizational justice can help explain the reasons behind quiet quitting based on the principles of reciprocity and fairness between employees and employers [11]. Social exchange theory stands as one of the most influential conceptual paradigms for comprehending employee behavior in the workplace [18]. This research is primarily grounded in social exchange theory, a widely applied framework for studying various organizational relationships, including those between employers and employees, as well as an employee’s relationship with the organization itself. Social exchange theory posits that interactions between employers and employees encompass both psychological and economic exchanges [9]. In this context, organizational support represents a supportive organizational resource, whereas work overload represents a job demand and change fatigue reflects strain associated with repeated organizational changes. Within these relationships, job satisfaction is considered a potential mediating mechanism between these workplace conditions and quiet quitting. There are several reasons why healthcare professionals opt for quiet quitting instead of resigning or retiring. The increasing cost of living and shrinking retirement portfolios, or insufficient retirement savings, have led to an increase in quiet resignation. Although financial rewards are not necessarily the main factor underlying employees’ enthusiasm for their work, recent workplace trends suggest that organizational expectations of employees may often be excessive [14].

### 1.1. Change Fatigue and Quiet Quitting

Change fatigue was described by researchers as “the repercussion of attempting to keep up with evidence-based practice due to the institution’s implementation strategies and timing without consideration of nursing workload” [19]. In the midst of a competitive environment, healthcare organizations encounter difficulties in both implementing and maintaining numerous changes aimed at promoting best practices and guaranteeing that healthcare providers deliver safe, competent care [20]. Changes in the workplace lead to fatigue in individuals, and empirical evidence suggests that there is a negative relationship between task changes and employees’ job satisfaction [21]. Previous research found that higher job satisfaction was significantly associated with lower change fatigue and greater resilience [22]. In another study, findings showed that change fatigue and job satisfaction were negatively correlated [23].

To the best of our knowledge, although previous studies have examined the relationship between change fatigue and job satisfaction, the literature directly addressing the relationship between change fatigue and quiet quitting remains limited. However, related research on organizational change processes and support for change suggests that change-related strain may contribute to employee withdrawal behaviors. More recent studies have also indicated that change fatigue may be associated with quiet quitting either directly or indirectly through factors such as workload and presenteeism [24]. Therefore, this study extends the existing literature by focusing specifically on the role of change fatigue in explaining quiet quitting behavior in the healthcare context.

### 1.2. Work Overload and Quiet Quitting

Actually, due to low wages, challenging shifts, and long working hours, COVID-19 has not been the source of change but rather acted as a catalyst, accelerating the pace of change [5]. HCWs who prefer to remain in the healthcare sector may seek alternative career options, either within their current profession or in another field, that offer shorter working hours and a lighter workload [3]. Quiet quitting may stem from various factors, including inadequate compensation, overwhelming workloads leading to burnout, stagnation in career progression, diminished intrinsic drive, and imbalance between work and personal life [25]. The studies provide empirical evidence that workload is a determinant of quiet quitting [3]. Boy and Sürmeli [3] indicated that job and wage inequities, workload, and role conflicts lead to burnout, turnover intention, and disengagement. The toxic organizational culture formed by behaviors such as burnout, turnover intention, and disengagement particularly diminishes the quality of healthcare services provided by young employees [26].

### 1.3. Organizational Support and Quiet Quitting

Perceived organizational support, as elucidated by social exchange theory, underpins the manager-employee dynamic. Inadequate support from the organization may lead employees to quiet quitting [25]. Since the concept of quiet quitting is relatively new in the literature, the relationship between quiet quitting and organizational support has not yet been sufficiently examined. However, it is known that organizational support exhibits a negative relationship with variables such as turnover intention [27]. Moreover, turnover intention is also related to quiet quitting. On the other hand, a toxic workplace environment is positively related to quiet quitting [28]. In the present study, examining the associations of change fatigue, work overload, and organizational support with quiet quitting may provide additional insights into the existing literature.

### 1.4. Job Satisfaction as a Mediator

Many employees feel they are underemployed, stuck in endless tasks, and underpaid for their worth. However, the low level of employee satisfaction is also often overlooked as another significant reason for exhibiting quiet quitting behavior [6]. Job satisfaction may provide a potential pathway linking workplace conditions, including work overload, organizational support, and change fatigue, with quiet quitting. As an indicator of how positively employees evaluate their work and work environment, job satisfaction may be relevant to their level of commitment and willingness to contribute beyond basic job requirements [9]. This mediating variable can provide insights into the psychological mechanisms by which antecedents are associated with quiet quitting. A study conducted by Galanis, Katsiroumpa, et al. [10] indicated a significant association between quiet quitting and job satisfaction. Based on the results of the study, high levels of quiet quitting were associated with low levels of job satisfaction. Research involving nurses has also shown that the positive relationship between psychological capital and work engagement was partly explained by job satisfaction [29]. Moreover, job satisfaction served as a mediator in the relationship between workload and quality of nursing care. Additionally, workload and job satisfaction are negatively correlated [30]. A mediation model could help us to analyze the complex psychological processes involved in quiet quitting.

Empirically, job satisfaction has been shown to operate as both an outcome and a process variable within organizational research. Prior studies demonstrate its mediating role between job-related antecedents (e.g., workload, organizational support) and positive outcomes such as work engagement and quality of care, indicating its capacity to transmit the effects of workplace conditions into behavioral responses [30,31,32,33]. At the same time, job satisfaction has been identified as both an outcome of organizational support and a predictor of withdrawal-related attitudes and behaviors, including those conceptually aligned with quiet quitting [34,35]. This dual positioning in the literature supports its relevance as a key intervening mechanism.

Accordingly, this study aims to bridge this gap by addressing the limited attention given to job satisfaction as an explanatory mechanism linking change fatigue to quiet quitting. While prior research has predominantly focused on direct negative outcomes of change fatigue or examined job satisfaction in isolation, this study integrates these perspectives by positioning job satisfaction as a central mediating pathway through which organizational antecedents are translated into quiet quitting behavior. In doing so, the present study contributes to the literature by offering a more comprehensive understanding of the psychological mechanism underlying quiet quitting, particularly in the healthcare context.

### 1.5. Present Study

Building on the theoretical and empirical background presented above, this study examines the relationships between change fatigue, organizational support, work overload, job satisfaction, and quiet quitting among healthcare professionals. Although previous research has separately addressed these variables [30,31,32,33], their relationships with quiet quitting, including the potential indirect role of job satisfaction, remain insufficiently examined together within the same healthcare study.

Rather than leaving their jobs or professions, employees who engage in quiet quitting tend to limit the effort and performance they contribute at work [6]. In practice, these employees tend to restrict their efforts to essential job duties, avoid taking on additional tasks, refrain from contributing new ideas, and generally work only within their scheduled hours [17]. To the best of our knowledge, the mediating role of job satisfaction in the associations of change fatigue, organizational support, and work overload with quiet quitting has received limited attention among healthcare workers. Considering the gaps mentioned above, this study aims to explore the mediating role of job satisfaction in the relationship between change fatigue, organizational support, and work overload and quiet quitting among healthcare professionals. Although change fatigue, organizational support, and work overload may theoretically be interrelated, the present study examines each antecedent separately in relation to quiet quitting, with job satisfaction positioned as the proposed mediating variable. This analytical approach was adopted to examine the hypothesized indirect association corresponding to each antecedent rather than to compare their mutually adjusted unique contributions.

**Figure 1:**
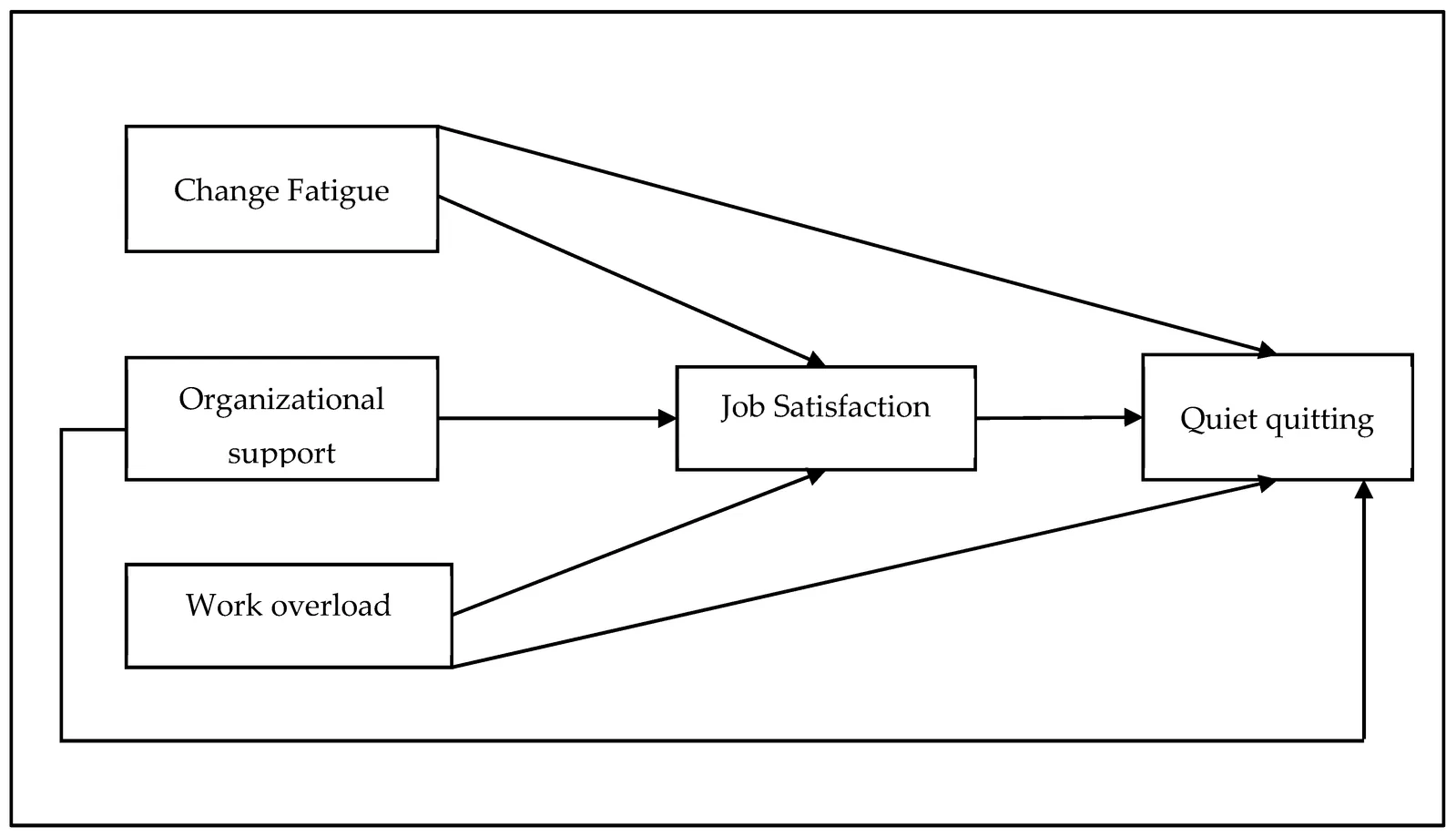
Conceptual model of the hypothesized associations among change fatigue, organizational support, work overload, job satisfaction, and quiet quitting.

Based on the theoretical and empirical background outlined above, the following hypotheses were formulated (Figure 1):

**H1.**  *Change fatigue is negatively associated with job satisfaction.*

**H2.**  *Organizational support is positively associated with job satisfaction.*

**H3.**  *Work overload is negatively associated with job satisfaction.*

**H4.**  *Change fatigue is positively associated with quiet quitting.*

**H5.**  *Organizational support is negatively associated with quiet quitting.*

**H6.**  *Work overload is positively associated with quiet quitting.*

**H7.**  *Job satisfaction is negatively associated with quiet quitting.*

**H8.**  *Change fatigue is indirectly associated with quiet quitting through job satisfaction.*

**H9.**  *Organizational support is indirectly associated with quiet quitting through job satisfaction.*

**H10.**  *Work overload is indirectly associated with quiet quitting through job satisfaction.*

## 2. Method

### 2.1. Participants

The study sample consisted of 487 healthcare workers. The sociodemographic and professional characteristics of the participants are reported in Table 1. Among the participants, 60.6% comprised nurses, health officers, and midwives, 21.4% were doctors, and the remaining 18.1% included laboratory technicians, X-ray technicians, and other technical roles. The majority of participants were female (57.3%) and married (55%). A total of 39.2% of the participants had a bachelor’s degree. The largest proportion of participants worked in the internal medicine unit (32%). Regarding age distribution, 46% of the participants were between 31 and 40 years old. The mean age of participants was 32.80 ± 5.58. Among the participants, 46% worked on a day-duty basis, 51.7% had been employed at the hospital for five years or less, and 69.7% had ten years or less of professional experience. Additionally, 41.3% reported that their income was equal to their expenditure.

### 2.2. Measures

#### 2.2.1. Change Fatigue

The change fatigue scale was developed by Bernerth et al. [36]. Turkish validation conducted by Ekingen and Yıldız [37]. Health professionals rated 6 self-reported items on a 5-point Likert scale, ranging from 1 (Strongly disagree) to 5 (Strongly agree). A higher score indicates higher change fatigue. The Cronbach’s alpha internal consistency coefficient of the scale was reported as 0.90. In the present study, it was 0.92. A sample item is “It feels like we are always being asked to change something around here.”

#### 2.2.2. Organizational Support

Organizational support was assessed using the scale originally introduced by Eisenberger et al. [38]. Türe and Yıldırım [39] subsequently adapted the shortened version for use among healthcare workers in Türkiye. The measure comprised nine self-report items, each scored from 1 (“strongly disagree”) to 5 (“strongly agree”). The mean item score was calculated, with possible mean scores ranging from 1 to 5. After reverse-coding the fifth and eighth items, A higher score indicates higher organizational support by the management. The Cronbach’s alpha internal consistency coefficient of the scale was reported as 0.88. In the present study, it was also determined to be 0.88. The scale measures employees’ perceptions regarding the extent to which the organization values their contributions and cares about their well-being and needs. Example items include “The organization I work for is proud of my achievements” and “The organization I work for is willing to help me when I have a special request.” The items assess the extent to which employees feel that their contributions are appreciated by the organization and that attention is given to their needs and welfare.

#### 2.2.3. Work Overload

The Workload Scale was developed by Imoisili [40] and Turkish validity and reliability was conducted by Şirin and Yücel [41]. The scale consists of 5 self-reported items and has a single-factor structure. Items are rated on a 5-point Likert scale ranging from 1 (Strongly disagree) to 5 (Strongly agree). The mean item score was calculated, with possible mean scores ranging from 1 to 5. The Cronbach’s alpha coefficient of the scale was 0.91. In our study, it was determined to be 0.88. A higher mean score obtained from the scale indicates a high level of work overload. Sample item of the scale was “I often deal with more work than I should.”

#### 2.2.4. Job Satisfaction

The Job Satisfaction Scale was developed by Judge et al. [42], and Turkish validation was conducted by Başol and Çömlekçi [43]. The scale consists of 5 self-reported items rated on a 5-point Likert scale ranging from 1 (Strongly disagree) to 5 (Strongly agree). The mean item score was calculated, with possible mean scores ranging from 1 to 5. A sample item of the scale is “I am satisfied with my current job.” As the mean score obtained from the job satisfaction scale increases, employees’ levels of job satisfaction also increase. The Cronbach’s alpha of the scale was 0.92. In our study, it was 0.72. According to the criteria recommended by Alpar [44], this value indicates an acceptable level of reliability.

#### 2.2.5. Quiet Quitting

The Quiet Quitting Scale was developed by Anand et al. [8] and validated in Turkish by Yıldız et al. [45]. The scale consists of 7 self-reported items rated on a 5-point Likert scale ranging from 1 (Strongly disagree) to 5 (Strongly agree). The mean item score was calculated, with possible mean scores ranging from 1 to 5. A sample item of the scale is “I often avoid working more hours if there is no additional pay.” The Cronbach’s alpha of the scale was 0.82. A higher score obtained from the scale indicates a high level of quiet quitting. The Quiet Quitting Scale is a unidimensional, seven-item self-report measure that assesses respondents’ reported tendency to limit their work-related effort and contribution rather than extend their effort beyond what they perceive as required. Accordingly, in the present study, quiet quitting was operationalized as a self-reported tendency rather than as actual turnover or an objectively observed workplace behavior. Its validity in Türkiye was tested by the researchers via confirmatory factor analysis. Evidence supporting the one-factor structure of the scale was obtained from confirmatory factor analysis. The model yielded χ^2^/df = 2.413 and RMSEA = 0.054, together with CFI = 0.976, GFI = 0.981, and AGFI = 0.962, indicating satisfactory overall model fit. For the present data, Cronbach’s α was calculated as 0.80.

### 2.3. Procedure

Initially, the necessary permissions for using the scales in this study were obtained from the researchers via email. Subsequently, ethical approval was obtained from the Batman University Ethics Committee (date: 8 November 2023; decision: 2023/06-27). This study employed paper-based versions of the questionnaires for data collection. A packet of questionnaires, comprising informational questions and change fatigue, organizational support, work overload, job satisfaction, and quiet quitting scales, was distributed. Participants required approximately 15 min to complete the scales. A convenience sampling approach was employed. Healthcare professionals working at the study hospital were eligible to participate. No additional eligibility criterion based on professional role or length of employment was applied. Healthcare professionals working in the hospital were approached in person during working hours by the researchers. Supervisors and managers were not involved in participant recruitment or questionnaire distribution. Data were collected in January and February 2024 in a city located in the southeast of Türkiye. The 487 healthcare workers included in this study exceeded the suggested sample size of 115–285 for detecting indirect effects in mediation models [46]. A total of 600 healthcare professionals were invited to participate in the study; 487 healthcare professionals completed the questionnaire (response rate: 81.16%). The remaining 113 healthcare professionals did not provide consent to participate. No incomplete questionnaires were included in the final dataset. The total number of healthcare professionals eligible for participation at the study hospital was not available to the researchers. Participants who agreed to take part in the study provided informed consent and were assured of the confidentiality and anonymity of their responses.

### 2.4. Data Analysis

Data analyses were conducted by utilizing IBM SPSS Statistics, version 25. Prior to primary analysis, descriptive statistics, such as mean and standard deviation, were conducted. The normality assumption of the data was assessed by examining skewness and kurtosis scores, as detailed in the results Section 3. These assessments verified that the data adhered to a normal distribution, validating the suitability of utilizing parametric tests in this study [47]. Following this, a Pearson product-moment correlation analysis was performed to investigate the relationships among the study variables. Potential multicollinearity among the study variables was assessed using tolerance and variance inflation factor (VIF) statistics. Regression assumptions were further evaluated through inspection of standardized residual histograms, normal P–P plots, and plots of standardized residuals against standardized predicted values. Potentially influential observations were assessed using Cook’s distance. Construct validity was further examined using a five-factor confirmatory factor analysis (CFA) measurement model estimated with maximum likelihood estimation, with the items loading on their respective constructs. The adequacy of the measurement model was assessed from the χ^2^/df ratio together with the CFI, TLI, and RMSEA values. For each construct, CR and AVE were then obtained, while the distinction among constructs was assessed by contrasting √AVE values with their corresponding correlations. Subsequently, three separate simple mediation analyses were conducted using the PROCESS macro version 4.2 (Model 4) for SPSS. In each analysis, job satisfaction was specified as the mediator and quiet quitting as the outcome, while change fatigue, organizational support, and work overload were examined separately as the focal predictor. Thus, the three models examined the indirect associations of change fatigue, organizational support, and work overload with quiet quitting through job satisfaction. The analyses were conducted separately because the primary objective was to examine the hypothesized indirect association for each antecedent rather than to estimate or compare their mutually adjusted unique contributions. Statistical significance was set at *p* < 0.05, and indirect associations were estimated from 5000 bootstrap resamples with 95% percentile confidence intervals [48]. An indirect effect was considered statistically significant when its bootstrap confidence interval did not include zero.

## 3. Results

### 3.1. Preliminary Findings

Before assessing the mediation model, preliminary analyses were performed, including descriptive statistics, reliability assessments, checking assumptions of normal distribution, and assessing correlation coefficients. The normality assumption was assessed by examining skewness and kurtosis values, adhering to the guideline of ±2 recommended by George and Mallery [49] and Tabachnick et al. [47]. Since the skewness and kurtosis values fell within the recommended range, it was determined that the data met the assumption of normal distribution (see Table 2). Multicollinearity diagnostics indicated no evidence of problematic multicollinearity. Across the regression models examined for diagnostic purposes, tolerance values ranged from 0.588 to 0.827 and VIF values ranged from 1.210 to 1.701. Visual inspection of the residual histograms, normal P–P plots, and standardized residual-versus-predicted-value plots did not indicate substantial departures from residual normality, linearity, or homoscedasticity. Although a small number of relatively large standardized residuals were observed, Cook’s distance values were low (maximum = 0.053), indicating no highly influential observations. The five-factor measurement model demonstrated acceptable overall fit to the data, χ^2^(454) = 1117.67, *p* < 0.001, χ^2^/df = 2.46, CFI = 0.916, TLI = 0.908, and RMSEA = 0.055, 90% CI [0.051, 0.059]. Standardized factor loadings ranged from 0.27 to 0.88. Composite reliability was 0.75 for job satisfaction, 0.93 for change fatigue, 0.88 for organizational support, 0.89 for work overload, and 0.81 for quiet quitting. The corresponding AVE values were 0.40, 0.68, 0.47, 0.62, and 0.38, respectively. Thus, composite reliability was satisfactory across the constructs, whereas convergent validity was stronger for change fatigue and work overload and more limited for some constructs. The Fornell–Larcker criterion was not fully satisfied for all construct pairs, indicating that discriminant validity should be interpreted with caution. The Cronbach’s alpha coefficients for the scales were determined as 0.72 for job satisfaction, 0.92 for change fatigue, 0.88 for organizational support, 0.88 for work overload, and 0.80 for quiet quitting.

Regarding the correlation analysis, job satisfaction exhibited a significant negative correlation with change fatigue (r = −0.51; *p* < 0.05) and work overload (r = −0.29; *p* < 0.05). Additionally, quiet quitting exhibited a significant positive correlation with change fatigue (r = 0.67; *p* < 0.05) and work overload (r = 0.48; *p* < 0.05). Furthermore, organizational support is positively correlated with job satisfaction (r = 0.23; *p* < 0.05), and it demonstrated a negative correlation with quiet quitting (r = −0.44; *p* < 0.05). Moreover, change fatigue negatively correlated with organizational support (r = −0.40; *p* < 0.05), while it positively correlated with work overload (r = 0.45; *p* < 0.05). Organizational support negatively correlated with work overload (r = −0.28; *p* < 0.05). The correlation between job satisfaction and quiet quitting was negative and significant (r = −0.55; *p* < 0.05). Correlation analysis results and all preliminary findings are presented in Table 2.

### 3.2. Mediation Analyses

Three separate simple mediation analyses were conducted to examine the indirect associations of change fatigue, organizational support, and work overload with quiet quitting through job satisfaction. In each model, one antecedent was entered separately as the focal predictor (X), job satisfaction as the mediator (M), and quiet quitting as the outcome (Y). The three models were therefore estimated separately rather than simultaneously. Unstandardized coefficients for the mediator and outcome models are presented in Table 3, and the bootstrapped indirect effects are presented in Table 4.

In the first model, change fatigue was negatively associated with job satisfaction (β = −0.51, *p* < 0.001), with change fatigue explaining 26% of the variance in job satisfaction. In the outcome model, change fatigue was positively associated with quiet quitting (β = 0.52, *p* < 0.001), whereas job satisfaction was negatively associated with quiet quitting (β = −0.28, *p* < 0.001). The total association of change fatigue with quiet quitting was also statistically significant (B = 0.47, *SE* = 0.02, 95% CI [0.43, 0.52], β = 0.67, *p* < 0.001). The standardized indirect association of change fatigue with quiet quitting through job satisfaction was statistically significant (β = 0.14, *SE* = 0.01, 95% bootstrap CI [0.11, 0.17]), as the confidence interval did not include zero. The outcome model explained 51% of the variance in quiet quitting. Because the direct and indirect associations were both statistically significant and operated in the same direction, this pattern was consistent with complementary partial mediation. In the second model, organizational support was positively associated with job satisfaction (β = 0.23, *p* < 0.001), with organizational support explaining 5% of the variance in job satisfaction. In the outcome model, organizational support was negatively associated with quiet quitting (β = −0.33, *p* < 0.001), and job satisfaction was also negatively associated with quiet quitting (β = −0.47, *p* < 0.001). The total association of organizational support with quiet quitting was also statistically significant (B = −0.47, *SE* = 0.04, 95% CI [−0.56, −0.39], β = −0.45, *p* < 0.001). The standardized indirect association of organizational support with quiet quitting through job satisfaction was statistically significant (β = −0.11, *SE* = 0.02, 95% bootstrap CI [−0.15, −0.06]), as the confidence interval did not include zero. The outcome model explained 41% of the variance in quiet quitting. Because the direct and indirect associations were both statistically significant and operated in the same direction, this pattern was consistent with complementary partial mediation. In the third model, work overload was negatively associated with job satisfaction (β = −0.29, *p* < 0.001), with work overload explaining 8% of the variance in job satisfaction. In the outcome model, work overload was positively associated with quiet quitting (β = 0.35, *p* < 0.001), whereas job satisfaction was negatively associated with quiet quitting (β = −0.54, *p* < 0.001). The total association of work overload with quiet quitting was also statistically significant (B = 0.35, *SE* = 0.03, 95% CI [0.29, 0.40], β = 0.49, *p* < 0.001). The standardized indirect association of work overload with quiet quitting through job satisfaction was statistically significant (β = 0.13, *SE* = 0.01, 95% bootstrap CI [0.09, 0.17]), as the confidence interval did not include zero. The outcome model explained 23% of the variance in quiet quitting. Because the direct and indirect associations were both statistically significant and operated in the same direction, this pattern was consistent with complementary partial mediation (Table 4).

## 4. Discussions

The findings showed that change fatigue, organizational support, and work overload were associated with quiet quitting both directly and indirectly through job satisfaction, highlighting job satisfaction as a potentially relevant explanatory mechanism in these relationships.

The findings indicated that higher change fatigue was associated with greater quiet quitting tendencies among healthcare workers, with job satisfaction showing a significant indirect role in this relationship. To the best of our knowledge, research directly examining the relationship between change fatigue and quiet quitting remains limited. However, in a study conducted on nurses in Türkiye, it was determined that change fatigue increased burnout and turnover intention and decreased organizational commitment [50]. An integrative review conducted by Beaulieu et al. [20] indicated that change fatigue is associated with various emotional and behavioral outcomes in employees, including disinterest, passive acceptance, stress, frustration, indecisiveness, grief, quiet resistance, increased absenteeism, productivity loss, low morale, and a lack of trust in the employer. Additionally, change fatigue has been consistently associated with lower levels of job satisfaction in previous research [21,22,23]. Lower levels of job satisfaction may be associated with greater quiet quitting behavior, which is the tendency of employees to fulfill their duties with minimum effort and only perform mandatory tasks without physically leaving the job. From the perspective of social exchange theory, these findings may be interpreted as a perceived imbalance in the reciprocal exchange relationship between employees and the organization, whereby sustained organizational demands without adequate reciprocity (e.g., support, recognition, or resources) may lead employees to reduce their discretionary work effort. Employees experiencing change fatigue may exhibit reduced psychological and behavioral investment in their work, which may be associated with quiet quitting as a withdrawal response. One possible explanation is that change fatigue may deplete employees’ cognitive and emotional resources, which may be associated with lower job satisfaction and, in turn, greater withdrawal-oriented responses such as quiet quitting. In this context, addressing change fatigue may be relevant to efforts aimed at reducing quiet quitting tendencies.

Consistent with previous research linking work overload to unfavorable employee outcomes, higher work overload was associated with greater quiet quitting and lower job satisfaction, with job satisfaction showing a significant indirect role in the relationship between work overload and quiet quitting. Previous studies revealed that work overload negatively affects employee behavior. In a study conducted by Sasongko et al. [51], it was found that work overload decreases job satisfaction and increases turnover intention. A study conducted on healthcare workers during the COVID-19 period revealed that workload was positively associated with burnout and turnover intention [52]. In another study, it was found that work overload negatively affected the emotional commitment of employees [53]. In addition, many studies have found that work overload reduces job satisfaction [54,55,56]. Work overload has been linked to persistent work pressure and increased stress among employees. This situation has been associated with lower levels of job-related motivation and higher levels of withdrawal-oriented attitudes. From a social exchange theory perspective, one possible explanation is that excessive workload without corresponding organizational reciprocity (e.g., support, recognition, or adequate resources) may create a perceived imbalance in the employee–organization relationship and may be associated with reduced discretionary effort. Sustained work overload may also be associated with lower job satisfaction, which may in turn be related to withdrawal-oriented responses such as quiet quitting.

Higher perceived organizational support was associated with lower quiet quitting and higher job satisfaction, with job satisfaction showing a significant indirect role in the relationship between organizational support and quiet quitting. These findings are broadly consistent with previous research linking organizational support with job satisfaction and withdrawal-related outcomes. Although research directly examining perceived organizational support in relation to quiet quitting remains limited, Xueyun et al. [9] found that perceived organizational support was indirectly associated with quiet quitting through employee well-being and job burnout. In a systematic review study conducted by Galanis, Moisoglou, Papathanasiou, et al. [27], it was found that organizational support negatively affects turnover intention. In a study conducted by Nadeem et al. [57], it was determined that perceived organizational support enhances employees’ positive evaluation of their work environment and increases job satisfaction while reducing withdrawal-related attitudes. Accordingly, it can be said that employees are less likely to consider leaving their jobs when they feel organizational support. Indeed, this situation is conceptually linked to quiet quitting as both reflect withdrawal-oriented responses within the employment relationship, although they represent distinct constructs. Indeed, in a study conducted by Galanis et al. [58] on nurses, it was found that turnover intention is positively associated with quiet quitting behavior. Similar to the findings in our study, previous research has found that organizational support increases job satisfaction [59,60]. These findings suggest that when employees perceive that their contributions are recognized and their well-being is valued, they are less likely to psychologically disengage from their work, even if they remain formally employed. Such perceptions may shape employees’ willingness to invest discretionary effort in their roles, thereby reducing tendencies toward minimal-effort work behaviors such as quiet quitting. In this sense, organizational support appears to operate as an important contextual factor that shapes employees’ attitudinal and behavioral responses through their overall evaluation of the work environment. One possible explanation is that greater perceived organizational support may contribute to more positive evaluations of the work environment and higher job satisfaction, which may in turn be associated with lower quiet quitting tendencies. Therefore, it can be said that increasing the support provided by organizations to employees may play an important role in addressing negative behaviors such as quiet quitting by enhancing employee job satisfaction.

Taken together, the findings suggest that job satisfaction may represent an attitudinal pathway linking workplace conditions with quiet quitting. Within the framework of social exchange theory, employees’ evaluations of organizational demands and resources may be reflected in their level of job satisfaction, which may in turn be associated with their willingness to invest discretionary effort at work. However, because the present study was cross-sectional, this interpretation should be regarded as a theoretically plausible explanation rather than evidence of a causal mechanism.

## 5. Implications and Limitations

The present study has several important implications for organizational management, particularly in the healthcare sector. Understanding the factors that are associated with quiet quitting can help managers develop strategies to mitigate its impact and enhance overall employee well-being and productivity. The study indicated that change fatigue is significantly associated with quiet quitting. This suggests that organizations should prioritize managing change effectively to reduce employee fatigue. Implementing structured change management processes, providing clear communication about changes, and offering support and resources during transitions may help address the negative effects of change fatigue. Such practices may also be relevant to supporting job satisfaction and reducing quiet quitting tendencies. Additionally, the positive association between work overload and quiet quitting suggests that workload management may be relevant when addressing quiet quitting. Organizations may consider strategies to balance workload, such as ensuring adequate staffing, distributing tasks more effectively, and promoting a healthy work–life balance. Such strategies may help support job satisfaction and may be relevant to reducing quiet quitting tendencies. Moreover, the study highlights the importance of perceived organizational support in relation to quiet quitting behavior. Organizations should foster a supportive work environment where employees feel valued and supported. This can be accomplished by implementing recognition programs, offering professional development opportunities, providing mental health resources, and ensuring that managers are approachable and attentive to employee needs. Greater organizational support was associated with lower quiet quitting and higher job satisfaction in the present study. Furthermore, job satisfaction plays a significant mediating role between change fatigue, organizational support, work overload, and quiet quitting. Organizations should prioritize enhancing job satisfaction by addressing the factors mentioned earlier. Cultivating a positive work culture, providing competitive compensation and benefits, and ensuring that employees find meaning and purpose in their work can greatly boost job satisfaction. Higher job satisfaction was associated with lower quiet quitting in the present study.

While the present study provides valuable insights into the mediating role of job satisfaction in the relationship between change fatigue, organizational support, work overload, and quiet quitting, there are several limitations that should be acknowledged in other studies. First, this study used a cross-sectional design, which limits the ability to understand causality between the variables. Longitudinal studies are necessary to establish causal relationships and to understand how these dynamics change over time. Second, all study variables were assessed using self-report measures collected from the same respondents at the same time, which may have introduced common method bias and social desirability bias. Some measurement-related limitations should also be acknowledged. Although composite reliability was satisfactory for all constructs, AVE was below 0.50 for some measures, and the Fornell–Larcker criterion was not fully satisfied for all construct pairs. In particular, one item of the Job Satisfaction Scale showed a relatively low factor loading. These findings suggest that convergent and discriminant validity were not uniformly strong in the present sample and should be considered when interpreting the observed associations. Future studies should further evaluate the measurement properties of these constructs in independent healthcare samples. Participants might overestimate positive behaviors or underestimate negative ones, affecting the accuracy of the findings. Third, the sample consists of healthcare workers, which may limit the generalizability of the findings to other industries or sectors. In addition, a convenience sampling approach was employed, and data were collected from healthcare professionals working in a single hospital located in one city in southeastern Türkiye. In addition, the three mediation models were estimated separately; therefore, the reported associations do not represent the unique effects of each antecedent after mutual adjustment for the other antecedents. Future studies may examine these variables within a simultaneous multivariable or structural equation modeling framework to distinguish their shared and unique contributions. Furthermore, the sample showed an unbalanced gender distribution, with women comprising a larger proportion of participants than men, which may limit the extent to which the findings reflect healthcare workers with different gender distributions. Therefore, the representativeness of the sample cannot be fully ensured, and caution should be exercised when generalizing the findings to healthcare workers in different regions, institutions, gender compositions, or healthcare systems. Various work environments may have distinct factors affecting job satisfaction and quiet quitting. Therefore, further research is required to explore these relationships in different settings using more diverse and balanced samples and, where possible, multicenter sampling designs. Additionally, the study was conducted within a specific cultural or national context that could restrict the generalizability of the findings to other cultural settings. Cultural influences can have a profound impact on work behaviors and attitudes, underscoring the importance of conducting cross-cultural studies to confirm these findings across diverse cultural contexts. Another limitation is that the study did not examine whether quiet quitting and its antecedents differed across professional groups, such as physicians, nurses, and other healthcare professionals. In addition, the mediation models were not adjusted for sociodemographic or occupational characteristics such as age, sex, marital status, professional role, shift type, professional experience, or organizational tenure; therefore, potential confounding by these characteristics cannot be excluded. Future studies should examine the robustness of the observed associations using theory-informed, prespecified covariate sets. Differences in occupational roles, job demands, responsibilities, and working conditions may influence how healthcare professionals experience the factors associated with quiet quitting. Similarly, years of professional experience and organizational tenure were not examined as potential factors associated with quiet quitting. Employees at different career stages or with different levels of organizational tenure may perceive and respond differently to workplace conditions. Consequently, the observed relationships should not be assumed to be uniform across professional groups or levels of experience and tenure. Future studies could examine these potential differences through subgroup comparisons, moderation analyses, or multigroup models. Lastly, other potential factors influencing quiet quitting, such as individual differences, organizational climate and culture, leadership style, and external stressors (e.g., economic conditions), were not investigated in this study. Incorporating these variables in future research could offer a more comprehensive understanding of quiet quitting. Overcoming these limitations in future studies can validate and expand upon the findings of this research, offering deeper insight into the factors associated with quiet quitting and the role of job satisfaction.

## 6. Conclusions

The findings of this study showed that change fatigue, organizational support, and work overload were significantly associated with job satisfaction and quiet quitting. Additionally, job satisfaction was significantly and negatively associated with quiet quitting. Furthermore, significant indirect associations of change fatigue, organizational support, and work overload with quiet quitting through job satisfaction were observed. These findings emphasize the potentially relevant role of job satisfaction in the associations between these workplace factors and quiet quitting.

To sum up, this study offers valuable insights into the factors associated with quiet quitting and the potential indirect role of job satisfaction among the healthcare workers included in the study. However, because of the cross-sectional design, the observed relationships should not be interpreted as evidence of temporal or causal relationships. Longitudinal and experimental studies are needed to examine the direction and potential causality of these relationships. In addition, because convenience sampling was used and the study was conducted in a single hospital in southeastern Türkiye, caution is warranted when generalizing the findings to other healthcare populations and settings.

## Figures and Tables

**Table 1:** Demographic characteristics of participants.

Variable	N	%	Mean ± SD
Gender			
Female	279	57.3	
Male	208	42.7	
Age			
24–30	207	42.5	32.80 ± 5.58
31–40	224	46.0	
≥41	56	11.5	
Marital status			
Single	219	45.0	
Married	268	55.0	
Education level			
High school	51	10.5	
Associate degree	124	25.5	
Bachelor’s degree	191	39.2	
Master’s degree	121	24.8	
Profession			
Nurse-Health Officer-Midwife	295	60.6	
Doctor	104	21.4	
Other	88	18.1	
Unit			
Internal medicine units	156	32.0	
Surgical units	112	23.0	
Intensive care unit	96	19.7	
Operating room	41	8.4	
Emergency room	82	16.8	
Type of shift			
Day shift	68	14.0	
On-call/duty shift	191	39.2	
Combined day and duty shifts	228	46.8	
Years of working at the hospital (years)			
≤5	252	51.7	13 ± 5.30
≥6	235	48.3	
Years of professional experience (years)			
≤10	339	69.6	20 ± 7.85
≥11	148	30.4	
Income Status			
More income than expenditure	96	19.7	
Income equals expenditure	201	41.3	
Less income than expenditure	190	39.0	
Total	487	100	

**Table 2:** Descriptive statistics and correlations between the study variables.

Variable	Mean	SD	Skew.	Kurt.	α	1	2	3	4	5
1. Change fatigue	3.51	0.90	−0.55	−0.07	0.92	1	−0.401 **	0.450 **	−0.516 **	0.675 **
2. Organizational support	2.92	0.60	0.06	−0.29	0.88		1	−0.282 **	0.236 **	−0.449 **
3. Work overload	3.37	0.89	−0.36	−0.58	0.88			1	−0.296 **	0.487 **
4. Job satisfaction	3.06	0.65	0.24	0.67	0.72				1	−0.556 **
5. Quiet quitting	2.49	0.63	−0.39	−0.25	0.80					1

Note: ** *p* < 0.01.

**Table 3:** Unstandardized regression coefficients for the three separate mediation models.

Antecedent	Consequent			
	M (Job Satisfaction)			
	B	*SE*	*t*	*p*
X1 (Change fatigue)	−0.37	0.02	−13.25	<0.001
Constant	4.35	0.10	43.09	<0.001
	R^2^ = 0.26			
	F = 175.81; *p* < 0.01			
X2 (Organizational support)	0.25	0.04	5.34	<0.001
Constant	2.31	0.14	16.25	<0.001
	R^2^ = 0.05			
	F = 28.58; *p* < 0.01			
X3 (Work overload)	−0.21	0.03	−6.82	<0.001
Constant	3.78	0.11	34.37	<0.001
	R^2^ = 0.08			
	F = 46.64; *p* < 0.01			
**Antecedent**	**Consequent**			
	**Y (Quiet quitting)**			
	**B**	***SE***	***t***	***p***
X1 (Change fatigue)	0.37	0.02	14.28	<0.001
M (Job satisfaction)	−0.27	0.03	−7.66	<0.001
Constant	2.03	0.17	11.56	<0.001
	R^2^ = 0.51			
	F = 256.02; *p* < 0.01			
X2 (Organizational support)	−0.35	0.03	−9.41	<0.001
M (Job satisfaction)	−0.46	0.03	−13.33	<0.001
Constant	4.93	0.13	36.45	<0.001
	R^2^ = 0.41			
	F = 172.34; *p* < 0.01			
X3 (Work overload)	0.25	0.02	9.76	<0.001
M (Job satisfaction)	−0.43	0.03	−12.48	<0.001
Constant	2.98	0.15	18.88	<0.001
	R^2^ = 0.23			
	F = 150.53; *p* < 0.01			

**Table 4:** Standardized indirect associations of change fatigue, organizational support, and work overload with quiet quitting through job satisfaction.

Paths					Standardized Indirect Effect (β)	BootSE	BootLLCI	BootULCI
Change fatigue	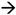	Job satisfaction	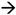	Quiet quitting	0.14	0.01	0.11	0.17
Organizational support	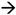	Job satisfaction	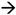	Quiet quitting	−0.11	0.02	−0.15	−0.06
Work overload	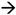	Job satisfaction	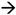	Quiet quitting	0.13	0.01	0.09	0.17

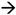
 indicates the hypothesized indirect pathway through job satisfaction.; BootSE: bootstrap standard error; BootLLCI: lower limit of the 95% bootstrap confidence interval; BootULCI: upper limit of the 95% bootstrap confidence interval. Statistical significance of an indirect association was determined by whether zero fell outside its bootstrapped 95% confidence interval.

## Data Availability

The data presented in this study are available on request from the corresponding author. The data are not publicly available due to privacy and confidentiality restrictions, as the dataset contains sensitive information collected from human participants.
